# Cancer-related mortality in Peru: Trends from 2003 to 2016

**DOI:** 10.1371/journal.pone.0228867

**Published:** 2020-02-06

**Authors:** Jessica H. Zafra-Tanaka, Janeth Tenorio-Mucha, David Villarreal-Zegarra, Rodrigo Carrillo-Larco, Antonio Bernabe-Ortiz

**Affiliations:** 1 CRONICAS Center of Excellence in Chronic Diseases, Universidad Peruana Cayetano Heredia, Lima, Peru; 2 Department of Epidemiology and Biostatistics, School of Public Health, Imperial College London, London, United Kingdom; 3 Centro de Estudios de Población, Universidad Católica los Ángeles de Chimbote (ULADECH), Chimbote, Peru; 4 Universidad Científica del Sur, Lima, Peru; Bielefeld University, GERMANY

## Abstract

**Objectives:**

In the last decade, Latin American (LA) countries, like Peru, have undergone an epidemiological transition that has changed the pattern of oncological cases. Given that Peru’s oncological pattern could illustrate those of other LA countries, we aimed at determining trends and changes in cancer-related mortality by age and sex in Peru between 2003 and 2016.

**Methods and results:**

A secondary data analysis using national deaths registries was conducted. Categories were created according to the 27 most frequent sites of presentation of cancer. We found that deaths attributed to cancer increased from 15.4% of all deaths in 2003 to 18.1% in 2016 (p<0.001). According to the cancer site, stomach cancer (19.1%) and lung cancer (11.5%) were the most frequent causes of death overall. In childhood (0 to 14 years), the two most frequent fatal cancers were leukemia (54.6% for boys and 53.5% for girls) and brain and nervous system tumors (19.4% for boys and 20.3% for girls). For teenagers and young male adults (15–49 years), stomach cancer (18.1%) and brain cancer (17.4%) were the leading causes of death; in their female counterparts, cervix uteri (20.0%) and breast cancer (16.1%) were the most mortal cancers. In adults (≥50 years), stomach (20.9% for men and 18.6% for women) and lung (12.7% for men and 10.4% for women) were the leading contributors to the burden of cancer deaths.

**Conclusions:**

Between the years 2003 and 2016, almost one fifth of deaths were attributed to cancer in Peru. Absolute and relative number of deaths due to cancer has increased in this period for both men and women; however, standardized mortality rates due to cancer have declined.

## Introduction

Globally, cancer represents the second leading cause of death and was responsible for 8.8 million of deaths in 2015 [1]. The largest cancer burden is in low- and middle-income countries (LMIC), where 60% of the total number of cases and 70% of the total number of deaths occur [2].

Several risk factors are associated with high cancer burden and mortality. Some of them can be non-modifiable such as age or sex [3, 4], while others are potentially modifiable such as nutrition and lifestyle behaviors [2]. The distribution of these factors plus the influence of demographic and epidemiological transitions have generated different patterns of cancer site presentations across countries and regions [5].

In the last decade, the oncological pattern has changed, and types of cancer once believed to be typical only of Western populations, such as colon or breast cancer, are now common in LMIC [2]. Two causes for these changes have been identified. First, behavioral risk factors for cancer such as unhealthy habits like smoking, fat and calorie-dense food consumption, and physical inactivity have increased in LMIC [6–8], and secondly, LMIC have experienced changes in population growth and ageing [9].

In the last decade, rates of oncological cases have increased in Peru. The oncological pattern in Peru, as well as in other countries in Latin America (LA), has changed from being mostly influenced by infectious agents to being affected by both infectious agents and lifestyles [5, 10]. Accordingly, between 2010 and 2012, the Lima surveillance system reported that largest mortality rates were due to prostate, stomach, and lung cancer for men; and stomach, cervix uteri and breast for women [11].

Prevention and early detection strategies should be tailored for a country or region [12]. Peru’s cancer mortality trends could provide an overview of the cancer transition in other LA countries, providing evidence to inform cancer control policies. Therefore, we aimed to determine the overall as well as age- and sex-specific trends in cancer-related mortality in Peru between 2003 and 2016 benefiting from nationally representative death registries.

## Methods

### Study design

A secondary data analysis using information of national deaths registries from 2003 to 2016 was conducted.

### Data sources

National death registries are usually collected by the Peruvian Ministry of Health (MINSA). We analyzed registries from 2003 to 2016. MINSA collects mortality data at the national level using different sources: 1) all health establishment records, 2) the Registro Nacional de Identificación y Estado Civil (RENIEC) and 3) the Ministerio Público [13–15].

Peruvian law dictates that when a person dies at any healthcare facility physicians are required to fill in a death certificate; these are compiled at the regional level and then sent to the MINSA central office. The health system in Peru is divided into Ministry of Health and Regional Government (MHRG), Social Security (EsSalud), Armed Forces and Police, and the Private sector; each sector offers differential treatment to their users. Information from MHRG are more likely to arrive at MINSA central office, and thus to be taken into account when managing mortality registries [16]. On the other hand, information from the remaining sectors might arrive late or not arrive at all, and may not be entirely reflected in national databases.

The Ministerio Público records and informs violent deaths: accidents, homicides or suspicious deaths (those not associated with previous medical conditions). However, data from Ministerio Público is underreported and do not always arrive at MINSA central office. Another source of vital record is RENIEC, which keeps records of vital events such as births and deaths nationally.

Given the multiple stages this information has to go through, there may be underreporting that needs to be taken into consideration when interpreting the results. Data quality control was performed excluding cases that presented inconsistent data (e.g. women with prostate cancer).

### Variables

In order to assess trends in mortality due to cancer, death registries in which the underlying cause of death started with the letter C according to the ICD-10 coding system were considered. We did not consider cases of cancer-related to HIV since the incidence of HIV is not high in Peru and cancer due to HIV accounts for a small proportion of deaths of people living with HIV [17].

**Fig 1:**
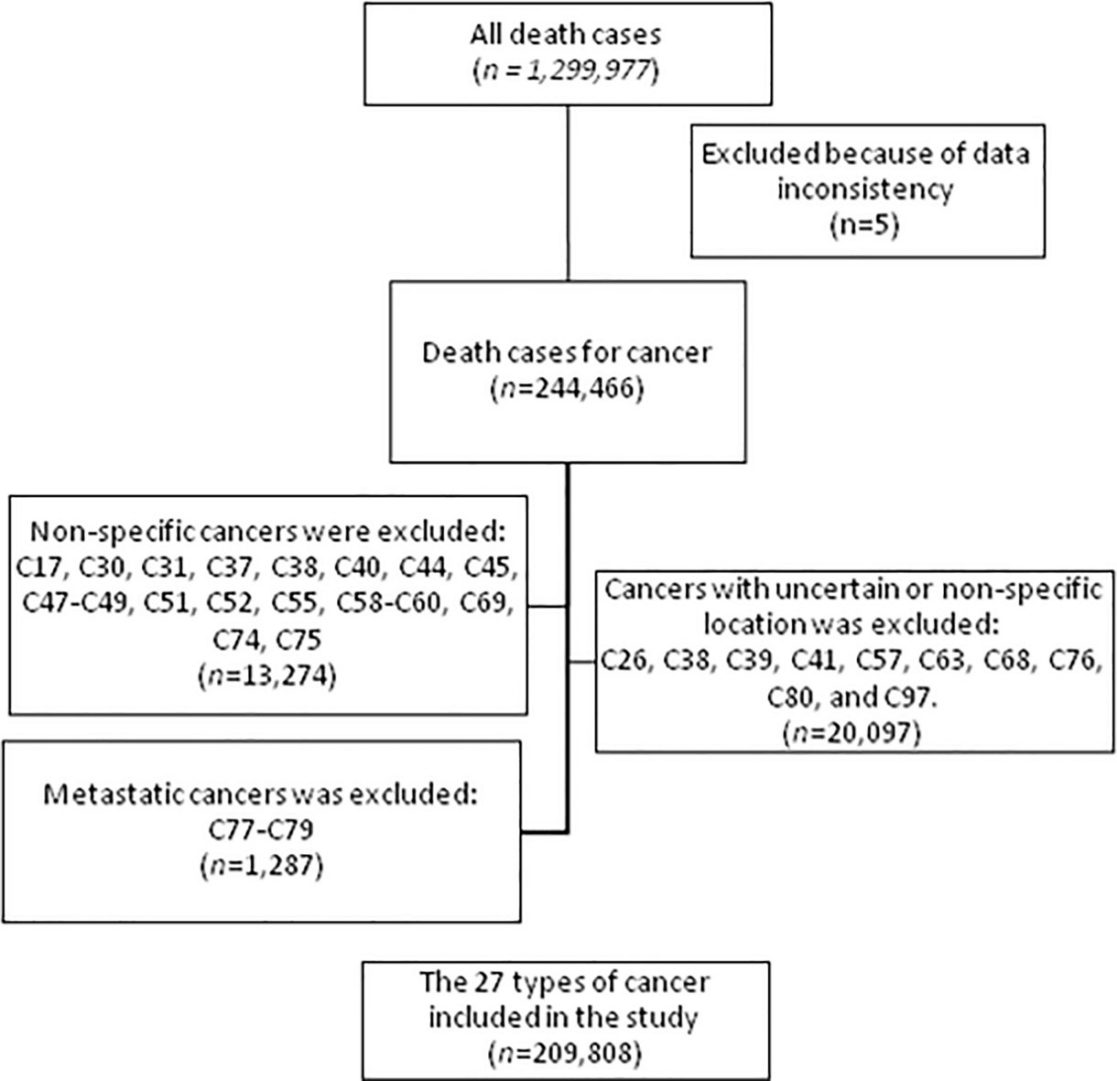
Flowchart of selected type of cancers included in the study.

Cancers of interest herein analyzed were the 27 most frequent types of cancers according to the GLOBOCAN [18]. We used the ICD-10 coding (only the two first code numbers) to classify the different types. The categories are as follows: lip and oral cavity (C00 –C08), nasopharynx (C11), other pharynx (C09, C10, C12-C14), esophagus (C15), stomach (C16), colorectum (C18 –C21), liver (C22), gallbladder (C23 –C24), pancreas (C25), larynx (C32), lung (C33 –C34), melanoma of skin (C43), Kaposi sarcoma (C46), breast (C50), cervix uteri (C53), corpus uteri (C54), ovary (C56), prostate (C61), testis (C62), kidney (C64 –C66), bladder (C67), brain and nervous system (C70 –C72), thyroid (C73), Hodgkin lymphoma (C81), non-Hodgkin lymphoma (C82 –C85 and C96), multiple myeloma (C88 and C90), and leukemia (C91 –C95). For the analyses, we excluded the following codes because they do not specifically address one site of presentation and were not included in GLOBOCAN:[18] C17, C30, C31, C37, C38, C40, C44, C45, C47-C49, C51, C52, C55, C58-C60, C69, C74, C75. We also excluded all cancer with uncertain or non-specific sites of location such as C26, C38, C39, C41, C57, C63, C68, C76, C80, and C97 (See Fig 1). Finally, cancers with ICD-10 codes ranging from C77 to C79 were not considered in the analyses because these represent metastatic cancer and a higher level of detail in the diagnosis and in the ICD-10 codes are needed to identify the primary site. Thus, to avoid misclassification, we decided not to include them.

We also considered sex and age at death. Age was categorized as follows: childhood (0 to 14 years), teenagers and young adults (15 to 49 years), and adults (≥50 years). According to the WHO and England statistics, the most common cancers vary by sex in these age groups [19, 20].

### Statistical methods

Absolute and relative frequencies were used to describe categorical variables, while central tendency and dispersion measures were used for quantitative variables. To compare and contrast the burden of deaths due to cancer in the previous years, we estimated mortality rates. We calculated the mortality rate dividing the number of deaths due to cancer by the expected population from the whole country for that year according to the National Institute of Statistics and Informatics (INEI in Spanish) [21]. These rates were age-standardized using the direct method and the global population 2000–2020 proposed by WHO [22]. Mann-Kendall test was used to assess time trends using cancer mortality age-standardized death rates. We also used a moving average technique using one lagged term, one forward term and the current observation to create smoother curves. Graphs were elaborated using Microsoft Excel 2016 and statistical analyses were carried out using Stata v.14 (Stata Corp, College Station, TX, US).

### Ethics

Data provided by MINSA is freely available and does not contain personal identifiers. Therefore, confidentiality and integrity were guaranteed.

## Results

### Trends in mortality due to cancer

Between 2003 and 2016, a total of 1 299 977 deaths occurred in Peru, of these, 244 471 (18.8%) were attributed to any cancer, and 209 813 (85.8% of all cancers) fall in one of the 27 categories used in this study (Fig 1). For those who died of cancer, the mean age at death was 65.4 years (SD = 18.5), and 52.9% were women.

The age-standardized mortality rates varied from 460.5 per 100 000 inhabitants in 2005 to 363.9 per 100 000 inhabitants in 2016, identifying a significant reduction over time (p < 0.001, p for moving average = 0.001). When analyzing deaths due to cancer, the age-standardized mortality rate varied from 90.9 per 100 000 inhabitants in 2005 to 66.0 per 100 000 inhabitants in 2016 (Table 1), identifying a significant reduction over time (p = 0.002, p for moving average < 0.001).

**Table 1:** Age-standardized cancer mortality rate.

Year	Age-standardized mortality rate per 100,000 hab. in Peru (Global population 2000–2020)	Age-standardized mortality rate due to cancer per 100,000 hab. in Peru (Global population 2000–2020)	Age-standardized mortality rate due to other causes per 100,000 hab. in Peru (Global population 2000–2020)	Age-standardized mortality rate per 100,000 hab. in Peru (Global population 2000–2020) for women	Age-standardized mortality rate due to cancer per 100,000 hab. in Peru (Global population 2000–2020) for women	Age-standardized mortality rate due to other causes per 100,000 hab. in Peru (Global population 2000–2020) for women	Age-standardized mortality rate per 100,000 hab. in Peru (Global population 2000–2020) for men	Age-standardized mortality rate due to cancer per 100,000 hab. in Peru (Global population 2000–2020) for men	Age-standardized mortality rate due to other causes per 100,000 hab. in Peru (Global population 2000–2020) for men
2005	460.5	90.9	369.7	402.4	90.9	311.4	527.7	92.8	434.9
2006	415.8	82.3	333.4	361.7	79.1	282.6	478.2	87.9	390.3
2007	429.8	83.5	346.4	373.2	82.8	290.4	495.6	85.9	409.7
2008	433.7	86.3	347.4	377.3	84.7	292.6	499.3	90.1	409.3
2009	440.2	84.8	355.5	382.3	83.3	299.0	507.2	88.1	419.0
2010	445.6	83.9	361.7	385.4	82.4	303.0	515.8	87.6	428.2
2011	421.9	80.2	341.7	376.8	83.2	293.7	480.2	81.1	399.1
2012	413.9	78.9	335.0	356.2	77.4	278.8	481.4	82.4	399.0
2013	403.8	76.4	327.4	349.7	75.0	274.7	467.1	80.1	387.0
2014	382.5	77.7	304.8	329.0	75.7	253.4	445.1	81.7	363.4
2015	369.8	74.6	295.2	318.2	72.4	245.7	430.2	78.9	351.3
2016	363.9	66.0	297.9	322.9	67.8	255.1	408.6	65.3	343.3

**Fig 2:**
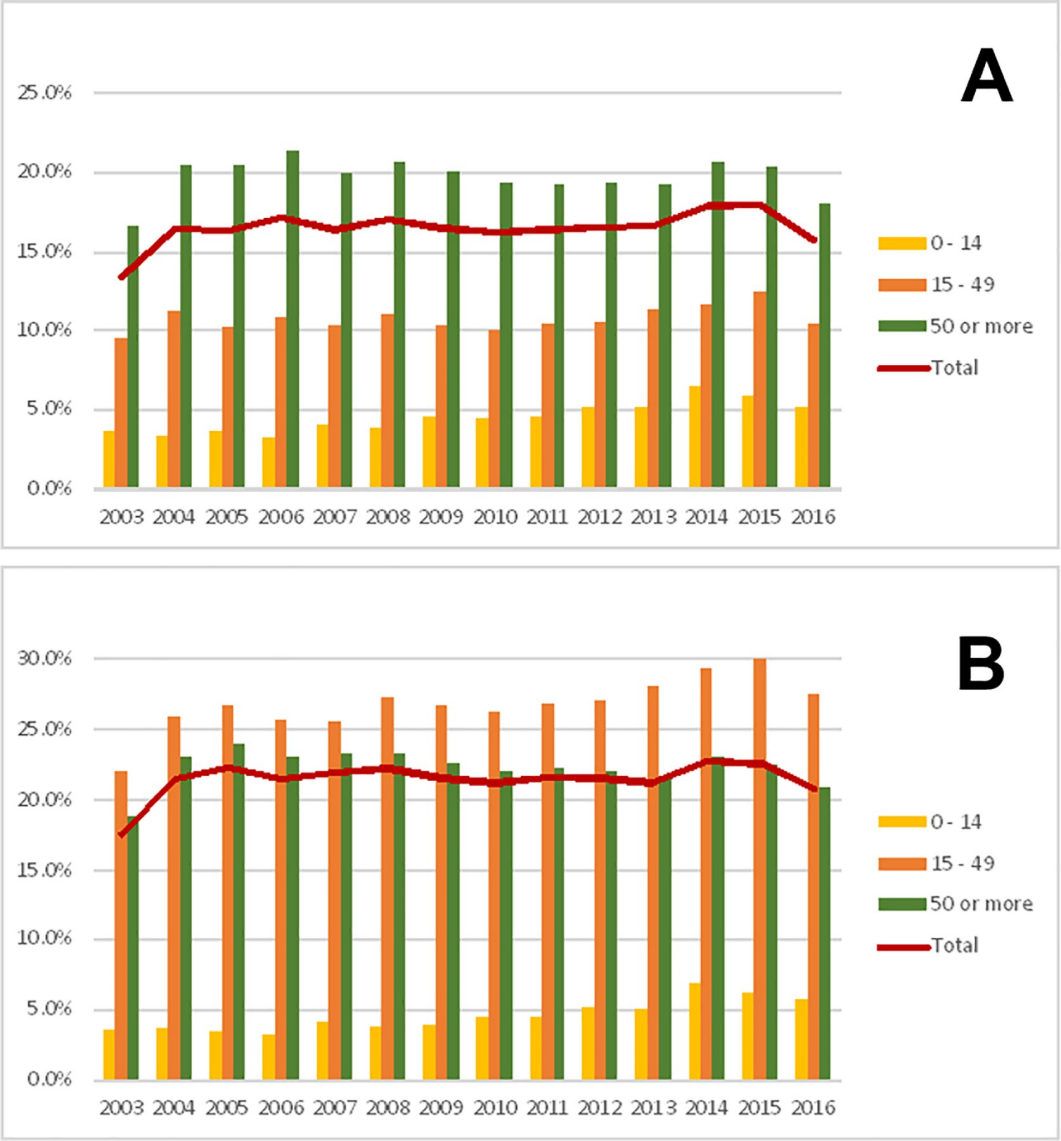
Cancer death trends across different age groups by sex: (A) Women and (B) Men.

Mortality trends for cancer are shown in Fig 2A and 2B for women and men separately. Overall, deaths attributed to any cancer increased from 15.4% of all deaths in 2003 to 18.1% in 2016 (p = 0.827, p for moving average = 0.029). These figures in women ranged from 13.1% to 20.8% (p = 0.080, p for moving average = 0.029), whereas in men these figures were 10.8% to 15.8% (p = 0.155, p for moving average = 0.029), respectively. For women, the age group that presented the highest proportion of deaths due to cancer was between 15–49 years; while for men, this group included those aged 50 years and above.

For women, the age-standardized mortality rate varied from 402.4 per 100 000 inhabitants in 2005 to 322.9 per 100 000 inhabitants in 2016, identifying a significant reduction in mortality trends over time (p = 0.014, p for moving average = 0.001). When analyzing deaths due to cancer in women, the age-standardized mortality rate varied from 90.9 per 100 000 inhabitants in 2005 to 67.8 per 100 000 inhabitants in 2016 (Table 1), a significant reduction in trend over time (p = 0.004, p for moving average < 0.001). For men, on the other hand, the age-standardized mortality rate varied from 527.7 per 100 000 inhabitants in 2005 to 408.6 per 100 000 inhabitants in 2016, identifying a significant reduction in trend over time (p = 0.001, p for moving average < 0.001). When analyzing deaths due to cancer in men, the age-standardized mortality rate varied from 92.8 per 100 000 inhabitants in 2005 to 65.3 per 100 000 inhabitants in 2016 (Table 1), a significant reduction in trend over time (p = 0.003, p for moving average < 0.001).

### Trends according to site of presentation

Stomach cancer (18.4%) and lung cancer (10.7%) were the most frequent causes of death overall (Appendix 1) across study years. Additionally, to depict variations across time, the ranking of the sex- and age-stratified top 10 cancers in 2003, 2009 and 2016 are shown in Appendix 2.

For teenagers and young adults between 2003 and 2016, stomach cancer ranked between the third and fourth place for women and first for men. In men and women ≥50 years, stomach cancer was the leading cause of cancer deaths in all years except in 2016, when prostate cancer was the leading cause of cancer death in men. Nevertheless, the proportion of deaths attributed to stomach cancer across time (2003–2016) has declined for those ≥50 years; from 24.4% to 16.44% among women (p = 0.006, p for moving average < 0.001), and from 25.7% to 21.8% among men (p = 0.038, p for moving average = 0.004).

Lung cancer was another leading cause of cancer death throughout the study period, especially among those ≥50 years. Between 2003 and 2009, the proportion of cancer deaths due to lung cancer increased from 8.7% to 10.6% for women and then level off through 2016.

### Trends according to site of presentation, age, and sex

**Fig 3:**
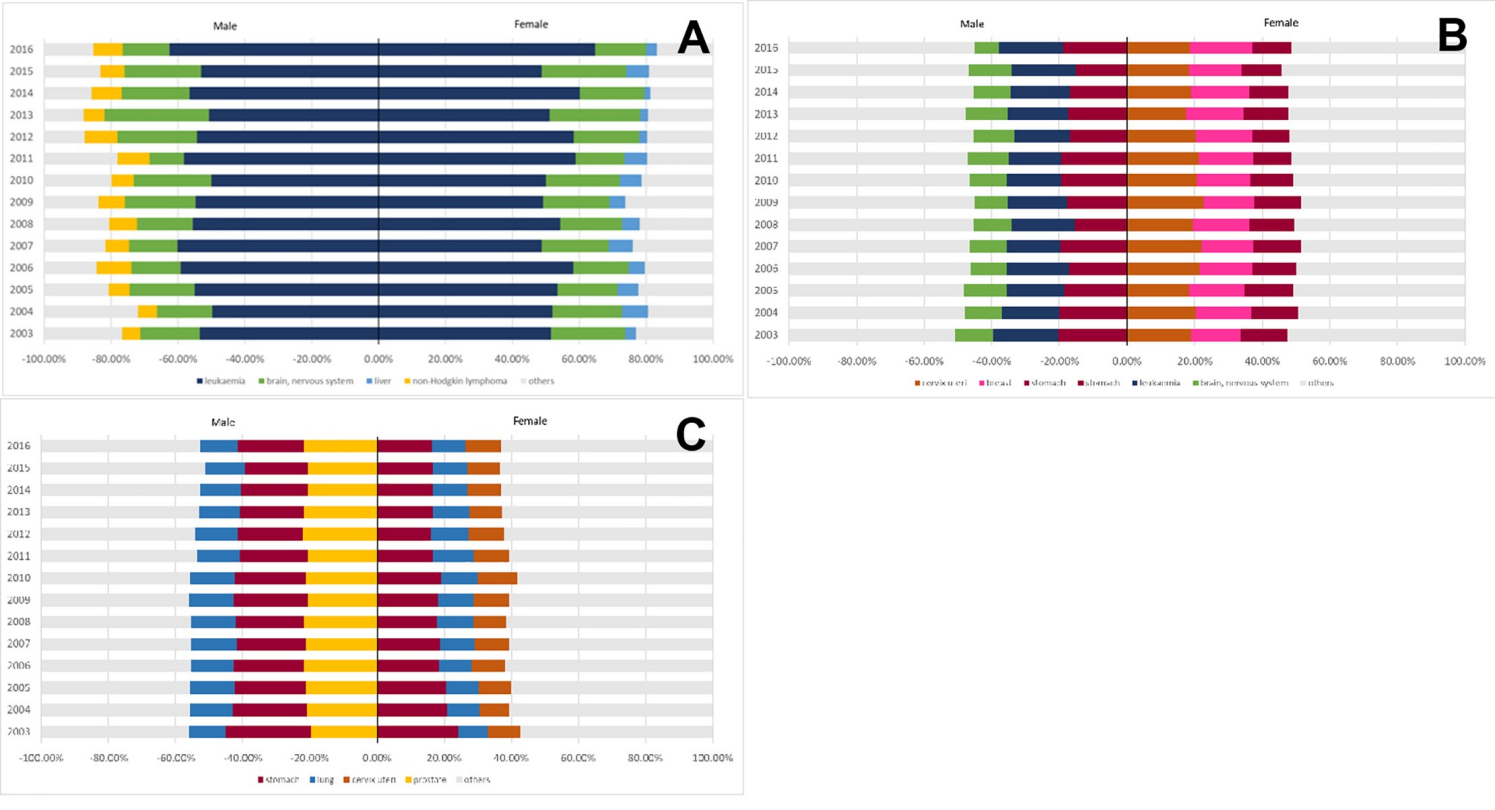
Cancer mortality, by major sites, in men and women, by age groups: (A) 0–14 years, (B) 15–49 years, and (C) 50 or more years.

Fig 3A, 3B and 3C display the percentages of the three leading causes of cancer deaths according to sex and age group. These figures also show their variations between 2003 and 2016. In childhood (0 to 14 years), the two leading causes of death were leukemia (55.6% for boys and 53.5% for girls) and brain and nervous system cancers (19.4% for boys and 20.3% for girls). For boys, the third most common cause of death was non-Hodgkin lymphoma (7.7%), while for girls it was liver cancer (5.1%).

For teenagers and young adults (15–49 years), stomach cancer (18.1%), brain cancer (17.4%), and non-Hodgkin lymphoma (11.5%) were the three leading causes of cancer death in men; while cervix uteri cancer (20.0%), breast cancer (16.1%), and stomach cancer (12.8%) were major contributors to cancer mortality in women.

In adults ≥50 years, stomach (20.9% for men and 18.6% for women) and lung (12.7% for men and 10.4% for women) were the leading contributors to the burden of cancer deaths. In addition, prostate and cervix uteri represented 21.0% and 10.1% of the deaths due to cancer in men and women, respectively.

## Discussion

Between 2003 and 2016 in Peru, almost one-fifth of deaths were attributed to cancer. Although age-standardized rates declined through these years, the absolute number of deaths due to cancer has increased in this period for both men and women, compared to deaths from other causes. The distribution of the neoplasm’s site presentation varied according to age and sex; however, the most important sites were stomach and lung for adults in general, and leukemia in children.

### Cancer mortality trends

Globally, deaths attributed to cancer have increased from 13.3% in the year 2000 to 15.5% in 2015 [19]. This can be explained by aging populations, lifestyle-related risk factors and a decrease in the proportion of deaths attributed to infectious diseases [19]. Our study found that one-fifth of deaths were attributed to cancer. WHO estimates of country-level causes of death, found a global estimate of 15.5% of deaths due to cancer, and a 20% estimate for upper-middle-income countries [19]. Given the current economic transition that LMIC undergo, lifestyle-related factors like, decreasing physical activity, increasing sedentary behaviors (e.g., due to changes in transportation), smoking and poor dietary habits are becoming more common in LMIC [5]. Given that these factors are present in Peru [23], it is not a surprise that cancer represents a significant cause of death in this country.

Overall, the age-standardized rate of cancer mortality seemed to have declined in Peru for both men and women from year 2005 to year 2016. This decline has also been found by a study from Our World in Data, that quantified the reduction of age-standardized rate of cancer mortality in Peru of 14.1%, which is higher than the reduction worldwide (7.2%) or the reduction in Andean Latin America (11.1%) for that period of time [24]. This may be explained by actions taken by the government to increase preventative and treatment services. For example, neoplastic care services have been decentralized and replaced with three regional institutes; increasing access to treatment. The government has also implemented new health strategies focused on cancer prevention, early detection, diagnosis, and proper treatment. Specifically, the Plan Esperanza [25] was implemented in 2012 with the objective to reduce cancer mortality and morbidity through better access to oncological care, promotion of healthy activities, prevention, and early diagnosis [25, 26], and it’s focused on breast, cervix uteri, colon, stomach, prostate, leukemia and lymphoma. Strategies implemented in HIC have demonstrated that prevention and early detection reduce cancer burden, however replicating these strategies in LMIC deal with healthcare infrastructure, lack of cancer awareness, insufficient funding, competing health priorities, and limited human resources [12].

One additional thing to note is that our study found a much higher reduction compared to that of the Our World in Data study; 27.4% versus 14.1%. This might be due to the types of cancer considered in the analysis; we have considered the 27 most frequent types of cancers and the data used by that study includes all cancer types [24]. A bigger reduction in the most frequent cancer is expected as public health interventions would be focused on those types of cancers.

When interpreting standardized-mortality rates is not possible to evaluate the influence of the aging of the population on mortality rates because of the age adjustment performed in the data. This is of great importance given that mortality is higher in older people, and this population is increasing in Peru. Thus, in 2005 people over 50 year represented only 14.8% of the entire population but in span of ten years this population increased to 18.3%. It is logical that when analyzing the proportion of deaths due to cancer, it has increased throughout the years for both men and women.

### Trends according to site of presentation, age, and sex

#### Childhood (0 to 14 years)

Leukemia was the most important cause of cancer deaths during childhood, followed by brain and nervous system cancer, as shown in different countries in previous studies [18, 27]. These types of cancers are more lethal in LMIC compared to high-income countries (HIC) [18]. Possible explanations are late diagnosis and abandonment of therapy. According to data of the CONCORD program, the inequities between HIC and LMIC could be reduced by improving the access to health care and providing appropriate treatment [28].

#### Teenagers and young adults (15 to 49 years)

Stomach, leukemia, and brain and nervous system cancer are the leading causes of cancer death among men between 15 and 49 years. In teenagers and young women; cervix uteri, breast and stomach cancer are the leading causes of death due to cancer. It is important to note that, within this age group, mortality due to cancer is much higher in women (around 20% in the studied years) compared to men (around 10% in the studied years), because of cervix uteri and breast cancer.

The cervix uteri cancer is the fourth most common cause of cancer death in women worldwide [2], 70% of the world's burden is in LMIC [2]. Our data ranked it as the leading cause of cancer death in women of this age. The low rates of survival could be attributed to late detection and lack of access to treatment. Recently, Peru has initiated a vaccination program against HPV in girls attending school [11, 29]. However, it has been only a few years since this program started, so the impact of the program cannot be seen yet.

Breast cancer is the most frequent cancer in women worldwide,[2] the incidence in LMIC doubles that of HIC [2]. In Peru, it is the most frequent cancer in women and our results ranked it as the second cause of cancer death in adult women. In LA, deaths are associated with late detection and treatment [30], which could also be the case in our country. For example, the proportion of in-situ breast cancer in LA countries rank between 4 to 6%, while for North America it is 17.3%, and the 5-year survival rates from 2010 to 2014 varied between 50.2% and 89.8% in LA, and from 88.2% to 90.2% in North America [31].

Stomach cancer is the third cause of death worldwide with high mortality rates seen in Central and South America [18]. In Peru, it represents the leading cancer cause of death in adults, and is possibly explained by Helicobacter pylori infections, dietary and lifestyle factors, and genetic factors [32]. There are reasons to believe that the trend of deaths from gastric cancer could be increasing in Peru because of waterborne transmission of H. pylori [32] due to poor hygiene, and antibiotic resistance to standard therapy for this bacteria [33–35]. Despite this, according to our analysis, the proportion of deaths due to gastric cancer has declined between 2003 and 2016.

#### Adults (≥50 years)

In adult men, stomach, prostate and lung cancer were the leading causes of death, whereas, in adult women, these cancers were stomach and lung cancer, followed by cervical cancer.

Lung cancer represents the second leading cause of death due to cancer. Worldwide, lung cancer is responsible for nearly one-fifth of deaths due to cancer according to GLOBOCAN [18]. Women have some advantages compared to men regarding lung cancer diagnosis and treatment: women are more likely to be diagnosed with a localized disease [36], and the response to chemotherapy is better in women [37, 38]. In spite of these advantages, lung cancer still poses a significant burden of mortality in women. This may be due to the difficulties in early detection and the lack of access to proper care that affects both sexes. Additionally, there are regional variations, which may be due to smoking and environmental exposures [10]. Even though the prevalence of daily smoking in Peru is still low (3.3%) [39, 40], it is still necessary to implement policies in order to further reduce tobacco use. Additionally, it is known that indoor smoke emissions are also associated with lung cancer [41]. Around the 20% of the Peruvian population still use biomass to cook, especially in rural areas [42], and if we consider poor living conditions, inefficient health and educational conditions–common in those areas–it represents a significant public health problem. Therefore, it is vital to promote strategies to improve cooking conditions in vulnerable populations.

Prostate cancer is the second leading cancer and the fifth leading cause of death from cancer in men worldwide. Among nations with high Human Development Index (HDI), prostate cancer incidence rates have increased due to the implementation of screening tests, whereas mortality rates have declined because of early detection and well-timed treatment [43–45]. Unfortunately, in our country, late-stage diagnosis in older men continues to be a problem, thereby increasing the lethality of these cancers.

### Limitations and strengths

Our study analyzed cancer mortality trends over more than a decade in Peru (2003–2016), and provides a view of how cancer mortality trends have changed over time. These trends are important not only to facilitate planning resource allocation but also to assess the impact of public health interventions.

Our study has some limitations, the most important being the lack of high-quality data to assess cancer mortality trends due to sub-registry errors in identification and codification of causes of deaths. When assessing the civil registration and vital statistics system in Peru, one study found that its performance was poor (<0.70) as it was around 57.3 between years 2005–2009, and around 49.9 between years 2010–2012 [46]. This might be due to the partitioned health registries that prevent the MINSA from getting all information in a timely manner [47], and registries from MHRG are more likely to have been reported and therefore are better represented than those from EsSalud or Armed Forces. Given that any Peruvian patient, independent of their type of health insurance coverage, can get health care in MHRG establishments; it is not possible for us to assess the level of under-registry from each sector.

Another limitation is the lack of measurement of certain variables that could help us to better understand our results. For example, some variables might be associated with access to health services such as socioeconomic status or whether each person had health insurance or not. Nonetheless, we feel that our study sheds important light on a significant public health concern in Peru: nationally representative cancer mortality trends.

## Conclusions

Between the years 2003 and 2016 in Peru, almost one-fifth of deaths were attributed to cancer. Both absolute and relative number of deaths attributed to cancer has increased in this period of time for both men and women; however, standardized mortality rates have declined. This could be a reflection of the health systems response to cancer care. However, cancer programs should be prepared for the increasing number of cases of cancer. Moreover, prevention, early detection and proper access to treatment strategies must consider differences in the mortality distribution of sites of neoplasm by age and sex.

## Supporting information

S1 Appendix (DOCX)

S2 Appendix (DOCX)

S1 Table Cancer deaths by sex and types of cancer in Peruvian population, 2003–2016. (DOCX)

S2 Table Ranking of the 10 leading types of cancers in 2003, 2009 and 2016, by sex and age group. (DOCX)
