# Correction: Cancer-related mortality in Peru: Trends from 2003 to 2016

**DOI:** 10.1371/journal.pone.0230271

**Published:** 2020-03-04

**Authors:** The PLOS ONE Staff

The fourth author's name is spelled incorrectly. The correct name is: Rodrigo M. Carrillo-Larco. The correct citation is: Zafra-Tanaka JH, Tenorio-Mucha J, Villarreal-Zegarra D, Carrillo-Larco RM, Bernabe-Ortiz A (2020) Cancer-related mortality in Peru: Trends from 2003 to 2016. PLoS ONE 15(2): e0228867. https://doi.org/10.1371/journal.pone.0228867

The publisher apologizes for the error.
