# Supplementary material for: Cancer-related mortality in Peru: Trends from 2003 to 2016
Source: PLoS One. 2020 Feb 6;15(2):e0228867. doi: 10.1371/journal.pone.0228867 (PMC7004369; doi:10.1371/journal.pone.0228867)
Supplement: S1 Table — (DOCX) [file pone.0228867.s003.docx]

# **S1 Table. Cancer deaths by sex and types of cancer in Peruvian population, 2003 - 2016**

| **Site of neoplasm** | **Overall** | **Female** | **Male** |
| --- | --- | --- | --- |
|  | **n (%)** | **n (%)** | **n (%)** |
| Stomach | 38511 (18.4) | 18396 (17.6) | 20115 (19.1) |
| Lung | 22459 (10.7) | 10397 (9.9) | 12062 (11.5) |
| Prostate | 18359 (8.8) | 0 (0.0) | 18359 (17.5) |
| Liver | 17251 (8.2) | 9230 (8.8) | 8021 (7.6) |
| Colorectum | 14977 (7.1) | 8112 (7.8) | 6865 (6.5) |
| Cervix uteri | 12755 (6.1) | 12755 (12.2) | 0 (0.0) |
| Breast | 12059 (5.7) | 11805 (11.3) | 254 (0.2) |
| Leukaemia | 11443 (5.5) | 5325 (5.1) | 6118 (5.8) |
| Pancreas | 9735 (4.6) | 5237 (5.0) | 4498 (4.3) |
| Non-Hodgkin lymphoma | 8858 (4.2) | 4118 (3.9) | 4740 (4.5) |
| Brain, nervous system | 8611 (4.1) | 3977 (3.8) | 4634 (4.4) |
| Gallbladder | 7829 (3.7) | 5302 (5.1) | 2527 (2.4) |
| Kidney | 4331 (2.1) | 162 (0.2) | 4169 (4.0) |
| Ovary | 4325 (2.1) | 1624 (1.6) | 2701 (2.6) |
| Multiple myeloma | 2731 (1.3) | 1105 (1.1) | 1626 (1.5) |
| Bladder | 2626 (1.3) | 1017 (1.0) | 1609 (1.5) |
| Oesophagus | 2566 (1.2) | 766 (0.7) | 1800 (1.7) |
| Thyroid | 1932 (0.9) | 1353 (1.3) | 579 (0.6) |
| Lip, oral cavity | 1887 (0.9) | 938 (0.9) | 949 (0.9) |
| Melanoma of skin | 1575 (0.8) | 740 (0.7) | 835 (0.8) |
| Larynx | 1197 (0.6) | 271 (0.3) | 926 (0.9) |
| Corpus uteri | 1131 (0.5) | 1131 (1.1) | 0 (0.0) |
| Other pharynx | 782 (0.4) | 348 (0.3) | 434 (0.4) |
| Hodgkin lymphoma | 757 (0.4) | 325 (0.3) | 432 (0.4) |
| Testis | 653 (0.3) | 0 (0.0) | 653 (0.6) |
| Kaposi sarcoma | 271 (0.1) | 105 (0.1) | 166 (0.2) |
| Nasopharynx | 197 (0.1) | 85 (0.1) | 112 (0.1) |
| Total | 209,808 | 104,624 | 105,184 |
