# Supplementary material for: Cancer-related mortality in Peru: Trends from 2003 to 2016
Source: PLoS One. 2020 Feb 6;15(2):e0228867. doi: 10.1371/journal.pone.0228867 (PMC7004369; doi:10.1371/journal.pone.0228867)
Supplement: S2 Table — (DOCX) [file pone.0228867.s004.docx]

# **S2 Table. Ranking of the 10 leading types of cancers in 2003, 2009 and 2016, by sex and age group**

| **Female 0 - 14 years** | | | | | | | | | | |
| --- | --- | --- | --- | --- | --- | --- | --- | --- | --- | --- |
| 2003 | | |  | 2009 | | |  | 2016 | | |
| Site of neoplasm | | (%) |  | Site of neoplasm | | (%) |  | Site of neoplasm | | (%) |
| 1 | Leukemia | 51.64 |  | 1 | Leukemia | 49.21 |  | 1 | Leukemia | 64.80 |
| 2 | Brain, nervous system | 22.13 |  | 2 | Brain, nervous system | 19.84 |  | 2 | Brain, nervous system | 15.20 |
| 3 | Lung | 5.74 |  | 3 | Liver | 4.76 |  | 3 | Kidney | 8.00 |
| 4 | Liver | 3.28 |  | 4 | Ovary | 4.76 |  | 4 | Liver | 3.20 |
| 5 | Non-Hodgkin lymphoma | 3.28 |  | 5 | Kidney | 4.76 |  | 5 | Non-Hodgkin lymphoma | 3.20 |
| 6 | Stomach | 2.46 |  | 6 | Non-Hodgkin lymphoma | 4.76 |  | 6 | Stomach | 1.60 |
| 7 | Cervix uteri | 2.46 |  | 7 | Lung | 3.17 |  | 7 | Lung | 1.60 |
| 8 | Gallbladder | 1.64 |  | 8 | Lip, oral cavity | 1.59 |  | 8 | Multiple myeloma | 1.60 |
| 9 | Ovary | 1.64 |  | 9 | Colorectum | 1.59 |  | 9 | Ovary | 0.80 |
| 10 | Kidney | 1.64 |  | 10 | Another pharynx | 0.79 |  | 10 |  |  |

| **Male 0 - 14 years** | | | | | | | | | | |
| --- | --- | --- | --- | --- | --- | --- | --- | --- | --- | --- |
| 2003 | | |  | 2009 | | |  | 2016 | | |
| Site of neoplasm | | (%) |  | Site of neoplasm | | (%) |  | Site of neoplasm | | (%) |
| 1 | Leukemia | 53.37 |  | 1 | Leukemia | 54.74 |  | 1 | Leukemia | 62.42 |
| 2 | Brain, nervous system | 17.79 |  | 2 | Brain, nervous system | 21.05 |  | 2 | Brain, nervous system | 14.09 |
| 3 | Liver | 7.36 |  | 3 | Non-Hodgkin lymphoma | 7.89 |  | 3 | Non-Hodgkin lymphoma | 8.72 |
| 4 | Stomach | 5.52 |  | 4 | Liver | 4.74 |  | 4 | Liver | 4.70 |
| 5 | Non-Hodgkin lymphoma | 5.52 |  | 5 | Lung | 3.16 |  | 5 | Kidney | 4.03 |
| 6 | Lung | 2.45 |  | 6 | Kidney | 2.63 |  | 6 | Testis | 2.68 |
| 7 | Prostate | 1.84 |  | 7 | Stomach | 1.58 |  | 7 | Stomach | 1.34 |
| 8 | Hodgkin lymphoma | 1.84 |  | 8 | Prostate | 1.05 |  | 8 | Multiple myeloma | 1.34 |
| 9 | Colorectum | 1.23 |  | 9 | Testis | 1.05 |  | 9 | Hodgkin lymphoma | 0.67 |
| 10 | Pancreas | 1.23 |  | 10 | Colorectum | 0.53 |  |  |  |  |

| **Female 15 - 49 years** | | | | | | | | | | | | | | | | | |
| --- | --- | --- | --- | --- | --- | --- | --- | --- | --- | --- | --- | --- | --- | --- | --- | --- | --- |
| 2003 | | |  | | 2009 | | | | | |  | 2016 | | | | | |
| Site of neoplasm | | (%) |  | | Site of neoplasm | | | (%) | | |  | Site of neoplasm | | | | (%) | |
| 1 | Cervix uteri | 18.83 |  | | 1 | Cervix uteri | | 22.66 | | |  | 1 | | Breast | | 18.57 | |
| 2 | Breast | 14.82 |  | | 2 | Breast | | 15.04 | | |  | 2 | | Cervix uteri | | 18.50 | |
| 3 | Stomach | 13.77 |  | | 3 | Stomach | | 13.67 | | |  | 3 | | Leukemia | | 12.43 | |
| 4 | Leukemia | 10.52 |  | | 4 | Leukemia | | 9.00 | | |  | 4 | | Stomach | | 11.67 | |
| 5 | Brain, nervous system | 7.36 |  | | 5 | Liver | | 6.46 | | |  | 5 | | Ovary | | 6.75 | |
| 6 | Lung | 5.93 |  | | 6 | Lung | | 6.11 | | |  | 6 | | Lung | | 5.68 | |
| 7 | Liver | 5.74 |  | | 7 | Brain, nervous system | | 5.84 | | |  | 7 | | Brain, nervous system | | 4.30 | |
| 8 | Ovary | 5.54 |  | | 8 | Ovary | | 4.81 | | |  | 8 | | Non-Hodgkin lymphoma | | 4.07 | |
| 9 | Non-Hodgkin lymphoma | 4.59 |  | | 9 | Colorectum | | 3.78 | | |  | 9 | | Colorectum | | 3.38 | |
| 10 | Colorectum | 4.49 |  | | 10 | Non-Hodgkin lymphoma | | 3.30 | | |  | 10 | | Liver | | 3.38 | |
| **Male 15 - 49 years** | | | | | | | | | | | | | | | | |  |
| 2003 | | |  | 2009 | | | | |  | 2016 | | | | | | |  |
| Site of neoplasm | | (%) |  | Site of neoplasm | | | (%) | |  | Site of neoplasm | | | | | (%) | |  |
| 1 | Stomach | 20.17 |  | 1 | | Stomach | 17.70 | |  | 1 | | | Leukemia | | 19.07 | |  |
| 2 | Leukemia | 19.34 |  | 2 | | Leukemia | 17.70 | |  | 2 | | | Stomach | | 18.95 | |  |
| 3 | Brain, nervous system | 11.46 |  | 3 | | Liver | 11.70 | |  | 3 | | | Non-Hodgkin lymphoma | | 9.42 | |  |
| 4 | Liver | 11.33 |  | 4 | | Lung | 10.52 | |  | 4 | | | Lung | | 9.19 | |  |
| 5 | Non-Hodgkin lymphoma | 9.12 |  | 5 | | Non-Hodgkin lymphoma | 10.52 | |  | 5 | | | Liver | | 7.91 | |  |
| 6 | Lung | 6.77 |  | 6 | | Brain, nervous system | 9.76 | |  | 6 | | | Brain, nervous system | | 7.09 | |  |
| 7 | Colorectum | 5.66 |  | 7 | | Colorectum | 6.33 | |  | 7 | | | Colorectum | | 6.28 | |  |
| 8 | Pancreas | 2.62 |  | 8 | | Pancreas | 2.90 | |  | 8 | | | Testis | | 4.65 | |  |
| 9 | Testis | 2.62 |  | 9 | | Testis | 2.58 | |  | 9 | | | Pancreas | | 2.56 | |  |
| 10 | Prostate | 1.52 |  | 10 | | Kidney | 1.72 | |  | 10 | | | Kidney | | 2.44 | |  |

| **Female 50 or more years** | | | | | | | | | | |
| --- | --- | --- | --- | --- | --- | --- | --- | --- | --- | --- |
| 2003 | | |  | 2009 | | |  | 2016 | | |
| Site of neoplasm | | (%) |  | Site of neoplasm | | (%) |  | Site of neoplasm | | (%) |
| 1 | Stomach | 24.38 |  | 1 | Stomach | 18.07 |  | 1 | Stomach | 16.44 |
| 2 | Cervix uteri | 9.73 |  | 2 | Cervix uteri | 10.87 |  | 2 | Cervix uteri | 10.52 |
| 3 | Liver | 9.70 |  | 3 | Lung | 10.61 |  | 3 | Breast | 10.42 |
| 4 | Breast | 8.78 |  | 4 | Liver | 9.68 |  | 4 | Lung | 9.99 |
| 5 | Lung | 8.68 |  | 5 | Breast | 9.40 |  | 5 | Colorectum | 8.80 |
| 6 | Colorectum | 7.55 |  | 6 | Colorectum | 8.58 |  | 6 | Liver | 7.69 |
| 7 | Gallbladder | 5.83 |  | 7 | Pancreas | 5.92 |  | 7 | Gallbladder | 6.04 |
| 8 | Pancreas | 4.70 |  | 8 | Gallbladder | 5.50 |  | 8 | Pancreas | 5.55 |
| 9 | Non-Hodgkin lymphoma | 3.48 |  | 9 | Ovary | 3.70 |  | 9 | Non-Hodgkin lymphoma | 4.30 |
| 10 | Leukemia | 3.48 |  | 10 | Non-Hodgkin lymphoma | 3.26 |  | 10 | Ovary | 4.07 |

| **Male 50 or more years** | | | | | | | | | | |
| --- | --- | --- | --- | --- | --- | --- | --- | --- | --- | --- |
| 2003 | | |  | 2009 | | |  | 2016 | | |
| Site of neoplasm | | (%) |  | Site of neoplasm | | (%) |  | Site of neoplasm | | (%) |
| 1 | Stomach | 25.67 |  | 1 | Stomach | 18.07 |  | 1 | Prostate | 21.79 |
| 2 | Prostate | 19.51 |  | 2 | Prostate | 10.87 |  | 2 | Stomach | 19.74 |
| 3 | Lung | 10.92 |  | 3 | Lung | 10.61 |  | 3 | Lung | 11.07 |
| 4 | Liver | 8.51 |  | 4 | Liver | 9.68 |  | 4 | Colorectum | 7.74 |
| 5 | Colorectum | 6.39 |  | 5 | Colorectum | 9.40 |  | 5 | Liver | 6.97 |
| 6 | Non-Hodgkin lymphoma | 4.20 |  | 6 | Pancreas | 8.58 |  | 6 | Non-Hodgkin lymphoma | 5.17 |
| 7 | Pancreas | 3.67 |  | 7 | Non-Hodgkin lymphoma | 5.92 |  | 7 | Pancreas | 4.88 |
| 8 | Leukemia | 3.12 |  | 8 | Kidney | 5.50 |  | 8 | Leukemia | 3.67 |
| 9 | Gallbladder | 3.10 |  | 9 | Leukemia | 3.70 |  | 9 | Gallbladder | 3.31 |
| 10 | Brain, nervous system | 2.82 |  | 10 | Brain, nervous system | 3.26 |  | 10 | Kidney | 2.86 |

*Totals do not add up to 100% as we show only the 10 most common cancers by age and sex group
